# Availability of Naloxone in Retail Pharmacies Following Introduction of Over-the-Counter Status

**DOI:** 10.1001/jamanetworkopen.2026.23617

**Published:** 2026-07-17

**Authors:** Lori Ann Eldridge, David Kline, Kayleigh Fields, Briana Lewis, James McMillian, Rachel L. Graves, Kathleen L. Egan

**Affiliations:** 1 Department of Health Education and Promotion, College of Health and Human Performance, East Carolina University, Greenville, North Carolina; 2 Department of Biostatistics and Data Science, Division of Public Health Sciences, Wake Forest University School of Medicine, Winston-Salem, North Carolina; 3 Department of Human Development and Family Sciences, College of Health and Human Performance, East Carolina University, Greenville, North Carolina; 4 Department of Emergency Medicine, Wake Forest University School of Medicine, Winston-Salem, North Carolina; 5 Department of Implementation Science, Division of Public Health Sciences, Wake Forest University School of Medicine, Winston-Salem, North Carolina

## Abstract

**Importance:**

Expanding access to naloxone is an essential strategy in addressing the overdose crisis in the US. In March 2023, the US Food and Drug Administration approved the first over-the-counter (OTC) naloxone nasal spray, representing a substantial shift in public health policy and pharmacy practice.

**Objective:**

To assess the same-day availability of naloxone without a prescription across US retail pharmacies and examine pharmacy and neighborhood factors associated with access following OTC approval.

**Design, Setting, and Participants:**

This cross-sectional study used a secret shopper design to assess availability of naloxone in retail pharmacies in the US from January 15 to April 30, 2024. Participants included a stratified random sample of 1108 pharmacies from the 7 largest corporate pharmacy chains and an eighth stratum incorporating other retail pharmacies.

**Exposures:**

Pharmacy characteristics (chain size, type, and affiliation) and neighborhood sociodemographic indicators (racial and ethnic composition and area deprivation index).

**Main Outcomes and Measures:**

Same-day naloxone availability without a prescription, location of naloxone within the store, price, and alternative access suggestions.

**Results:**

Among the 1108 pharmacies contacted, an estimated 61.40% (95% CI, 57.34%-65.46%) of those with naloxone in stock reported it was available without a prescription. Naloxone was located at the pharmacy counter (estimate, 58.14% [95% CI, 53.59%-62.68%]), self-service aisles (estimate, 31.74% [95% CI, 27.59%-35.89%]), and front checkout areas (estimate, 8.99% [95% CI, 6.64%-11.34%]). The mean (SE) cost was $52.07 ($1.50) (95% CI, $49.12-$55.02). Among pharmacies without same-day naloxone (estimate, 38.60% [95% CI, 34.54%-42.66%]), respondents equally referred callers to another pharmacy or had no suggestions (estimate, 45.84% [95% CI, 37.77%-53.91%]), 4.46% (95% CI, 0.70%-8.21%) offered other suggestions (hospital, public service agencies, online service, or cannabis dispensary), 3.87% (95% CI, 0.48%-7.25%) suggested the public health department, and 0.06% (95% CI, 0.00%-0.17%) suggested syringe service programs. Pharmacies classified as food and/or mass merchandisers, independent, or medical affiliated had a lower odds ratio of offering same-day naloxone compared with corporate chains. In separate analyses, pharmacies located in areas with higher proportions of White residents were more likely to offer naloxone. Area deprivation index–defined neighborhood disadvantage, which does not include race in its calculation, was not associated with naloxone availability.

**Conclusions and Relevance:**

In this cross-sectional study of pharmacies across the US, same-day naloxone availability and price remained uneven following OTC approval. As an initial national evaluation of same-day naloxone availability and price after OTC approval, the findings showed differences in implementation across pharmacy types and community demographic features. Although enactment of the OTC policy was necessary, its intended benefits were not realized equally in practice. Expanding pharmacist education and targeting support for independent and medical-affiliated pharmacies may help improve naloxone access, especially in rural and underserved areas.

## Key Points

**Question:**

What is the same-day availability of naloxone without a prescription across US retail pharmacies following its approval for over-the-counter sale?

**Findings:**

In this cross-sectional study of 1108 pharmacies, 61.4% of pharmacies reported same-day naloxone availability without a prescription, often located at the pharmacy counter, and a mean cost of $52.07. Corporate chains and communities with higher proportions of White residents were more likely to offer naloxone.

**Meaning:**

These findings suggest that naloxone access still depends on location and pharmacy type, highlighting the urgent need to make this medication affordable and available to everyone, everywhere.

## Teaser

This cross-sectional study assesses nationwide availability of naloxone, including its retail price, in-store placement, and variation by pharmacy type and geographic characteristics.

## Introduction

The overdose crisis continues to affect many populations across the US, with at least 1 person dying from an opioid overdose every 5 minutes.^1^ According to the US Centers for Disease Control and Prevention (CDC), 64.7% of drug overdose deaths have at least 1 potential opportunity for intervention.^2^ Although emerging evidence suggests that recent declines in overdose mortality may be partly attributable to changes in fentanyl availability, potency, or exposure risk, timely naloxone access remains essential because it is one of the few interventions that can reverse opioid toxicity at the point of overdose.^3^

One form of intervention to prevent overdose is administering naloxone in a timely manner. Naloxone is an opioid antagonist that reverses opioid overdose and can be administered by bystanders before emergency responders arrive. Because many overdose deaths involve potential opportunities for intervention, timely naloxone access remains a critical overdose prevention strategy.^4^ A recent study found that nearly 40% of opioid overdose deaths had a bystander who could have intervened and provided naloxone.^5^ It has been shown that communities with overdose education and naloxone distribution have lower rates of overdose mortality than communities without those programs.^6,7^ Thus, it is key to educate bystanders on how to obtain and administer naloxone.

Increasing access to naloxone is vital to combat the overdose epidemic. Pharmacies play a crucial role in naloxone access, with nearly 89% of US residents living within 5 miles and 50% within 1 mile of a pharmacy.^8^ Until March 2023, naloxone required a prescription; however, most states had standing orders or naloxone protocols allowing pharmacists to dispense naloxone without a prescription.^4^ While these standing orders played a vital role in increasing naloxone availability, barriers remained for pharmacists and the public. Due to varying laws across states, the public and pharmacists have reported confusion about naloxone access in pharmacies.^9,10^ Further, individuals who have requested naloxone from pharmacies report experiencing stigma.^11^

Research studies demonstrate that despite standing orders for naloxone, its availability remained uneven; for example, a systematic review^12^ published in 2022 found that 62.8% of pharmacies stocked naloxone, with significant variation across states and pharmacy types. Independent pharmacies were consistently less likely to stock naloxone than chain pharmacies. While the review found similarities in naloxone availability across rural and urban areas, there were persistent inequities in cost across the included studies, with higher prices in rural and suburban settings compared with urban ones. The average price for naloxone ranges from $120 to $150.^12^ From 2018 to 2022, there was a decrease in out-of-pocket costs for naloxone, but the cost of naloxone remained high for self-pay compared with filing with insurance.^13^

Following the launch of over-the-counter (OTC) naloxone in 2023, findings from North Carolina suggest modest improvements in naloxone availability and cost. The same-day availability increased from 79.2% in 2023 to 85.4% in 2024,^14^ and the mean out-of-pocket cost for cash-paying customers decreased from approximately $90.43 in 2023 to $62.94 in 2024.^14^ This reduction still leaves naloxone prices higher than the suggested retail price of $44.99 and higher than prices for insured individuals using prescriptions.^15^ A post-OTC approval study in North Carolina^16^ found that pharmacy type was associated with OTC access to naloxone. Of the 500 pharmacies contacted that sold naloxone, 57.4% stocked OTC naloxone, and most often, the naloxone was stocked behind the counter (62.7%). They found that rurality was not associated with stocking, cost, or sales of naloxone. However, the type of pharmacy did matter, as independent pharmacies were significantly less likely to sell or offer same-day pickup for naloxone or to place OTC naloxone on open shelves within the store compared with chain pharmacies.^16^ A 2025 study conducted in Connecticut^17^ found that nearly all pharmacies surveyed (stocked naloxone (151 [95.4%]), but the placement of naloxone varied. Most kept naloxone behind the counter (111 [73.5%]), with very few stocking it on an aisle shelf (29 [19.2%]). The results of this study were similar to the North Carolina studies, reporting that independent pharmacies’ prices were higher than those of the chain pharmacies.^17^ Seven months after OTC naloxone approval, a survey of pharmacists across Alabama, Georgia, Mississippi, North Carolina, and Tennessee found that 18 of 47 respondents (38.3%) reported their pharmacy stocked naloxone, with a median price of $50.00.^18^ Most respondents (95.3%) practiced in independent pharmacies. A study conducted in Kentucky, Massachusetts, New York, and Ohio^19^ found that OTC naloxone sales were almost exclusively Narcan nasal spray (99%), with only 2 stores per 100 000 people participating in the sale of OTC naloxone. Average prices of OTC naloxone were consistent across the states and were near the recommended retail price ($44.81).^19^

Despite early data on OTC naloxone sales, substantial gaps remain in understanding the availability, accessibility, and affordability of naloxone across the US. No prior research has evaluated these factors on a national scale following the March 2023 OTC approval. This study is the first, to our knowledge, to provide a nationwide assessment of naloxone availability, including its retail price, in-store placement, and variation by pharmacy type and geographic characteristics.

## Methods

### Study Design and Setting

We conducted a cross-sectional study with a telephone-based secret shopper design among a sample of pharmacies across the US from January 15 to April 30, 2024. Secret shopper designs are used to assess health care delivery to patients and community members objectively. The strength of this design lies in its ability to elicit realistic and unbiased perspectives on the patient experience.^20^ The study posed minimal risk and collected no identifiable caller information; therefore, informed consent was waived by the Wake Forest University School of Medicine Institutional Review Board, which deemed the study exempt. We used the Strengthening the Reporting of Observational Studies in Epidemiology (STROBE) guideline for cross-sectional studies to support complete and transparent reporting. The full details of the study design have been published elsewhere.^21^ The codebooks for the medication disposal program and same-day naloxone without a prescription reporting sheet are included in the eAppendix in Supplement 1.

### Study Sample

A comprehensive list of active retail pharmacies in the US was obtained in October 2023 from a paid directory from an organization that has closed since the study was conducted (Hayes Directories, Inc). This dataset included 56 451 pharmacies and provided key identifying information such as pharmacy name, street address, telephone and fax numbers, and an indicator for chain vs independent pharmacy status (a chain was defined as having ≥8 locations). Pharmacies were excluded from the sampling frame if they (1) provided only compounding services, (2) operated exclusively as mail-order or delivery pharmacies, (3) lacked a brick-and-mortar address, and (4) were confirmed to serve a veterinary-only clientele. While the Hayes Directory excluded online-only businesses, additional screening was required to identify and remove records with only a post office box and pharmacies without public access.

We classified pharmacies into corporate chains with more than 1000 store locations in the Hayes Directories list, including subsidiaries and brands (eg, some Walgreens locations operate as Duane Reade) to create 8 strata: (1) CVS Pharmacy Inc, (2) Walgreens, (3) Walmart Inc, (4) Rite Aid Corporation, (5) Kroger Co, (6) Albertsons Companies Inc, (7) Publix Super Markets Inc, and (8) other retail pharmacies. We conducted disproportionate stratified random sampling with these 8 strata to maximize the likelihood of sampling diverse corporations and independent pharmacies.

We sampled 125 pharmacies per stratum to meet our goal of collecting data from 1000 pharmacies. To account for potential nonresponse or ineligible pharmacies, we oversampled 25 pharmacies per strata, resulting in an initial sample of 1200 pharmacies.

### Data Collection

Three data collectors (ie, secret shoppers) were trained to call pharmacies posing as individuals who needed to obtain naloxone. Telephone calls were used to feasibly assess pharmacies nationwide and reflect how community members may check service availability before visiting. Secret shoppers followed a semistructured script to ask pharmacies about same-day naloxone. Training of the secret shoppers consisted of a 2-hour session in which data collectors learned the script, made practice calls to pharmacies not within the sampling frame (≤10 pharmacies per data collector), and agreed on standard responses to potential questions during a debrief. During and after each call, data collectors completed a secure REDCap (Research Electronic Data Capture; Vanderbilt University) survey about their experience with the pharmacy staff.

### Outcomes and Measures

The primary outcome was the same-day availability of naloxone without a prescription. Pharmacies were coded as offering same-day naloxone without a prescription if the pharmacist confirmed same-day availability and stated that no prescription was required. Additional measures collected through the secret shopper protocol included the location of naloxone within the store, referrals to other naloxone access points, and the out-of-pocket cost of naloxone.^21^

Pharmacies were classified as chains, food and/or mass merchandisers, independent, or medical affiliates. Pharmacies with 8 or more locations were labeled as chains, unless they were affiliated with a food store or mass merchandiser (eg, Wal-Mart, Kroger). Pharmacies with 1 to 7 locations were labeled as independent pharmacies. Pharmacies affiliated with hospital systems were coded as medical affiliates.

Neighborhood-level sociodemographic characteristics for sampled pharmacies were obtained from the US Census Bureau 2022 American Community Survey 5-year estimates, accessed through the Social Explorer application, and the 2022 area deprivation index (ADI) from the University of Wisconsin Neighborhood Atlas.^22^ Racial and ethnic composition (including American Indian or Alaska Native, Black or African American, Hispanic or Latino ethnicity of any race, and White race of any ethnicity) and neighborhood disadvantage were assessed continuously as separate measures. American Community Survey measures were collected at the census tract and block group levels. ADI scores were derived at the block group level and measured socioeconomic disadvantage based on income, educational attainment, employment, and housing quality; race is not included in the ADI calculation. These data were temporally similar to the data collection period.

### Statistical Analysis

A calculated nationally representative weighted estimate of same-day naloxone availability without a prescription was conducted. Sampling weights were applied to adjust for the disproportionate stratified random sampling design. Weights were derived based on the proportion of corporate pharmacies in the analytic sample relative to their representation in the full Hayes Directory population.

To examine pharmacy- and neighborhood-level factors associated with these outcomes, a logistic regression analysis was conducted that accounted for the stratified survey design and generated nationally representative estimates. We originally planned to use robust SEs to account for potential residual clustering by state, but they were not supported within the procedure used to account for the survey design. This is noted as a limitation. Analyses proceeded in 2 steps. First, we estimated the unadjusted association between each covariate and naloxone availability. The second step was intended to be a multivariable model but was not conducted due to lack of unadjusted associations. Analyses were performed using SAS, version 9.4 (SAS Institute Inc). Results are reported as odds ratios (ORs) with corresponding 95% CIs. Two-sided *P* < .05 indicated statistical significance.

## Results

We were able to contact 1108 pharmacies for our final analytic sample. A total of 91 of 1200 (7.58%) were not included in the final sample due to permanent store closures, not serving the public, or not answering the telephone call after 3 attempts. Secret shoppers spoke with the first pharmacy staff member who answered the telephone. This approach was used to reflect a consumer experience. If the staff person reported their role, it was recorded.

### Naloxone Availability at Retail Pharmacies Across the US

Same-day naloxone without a prescription was available at an estimated 61.40% (95% CI, 57.34%-65.46%) of locations, while it was unavailable at 38.60% (95% CI, 34.54%-42.66%). Among the pharmacies that had naloxone available the same day, an estimated 19.73% (95% CI, 15.74%-23.71%) indicated that a prescription was required to obtain naloxone that day.

For pharmacies with same-day naloxone availability, naloxone was most commonly located at the pharmacy counter (estimate, 58.14% [95% CI, 53.59%-62.68%]), followed by self-service areas (estimate, 31.74% [95% CI, 27.59%-35.89%]) and front checkout (estimate, 8.99%; [95% CI, 6.64%-11.34%]). A small proportion (<1%) was categorized as other or missing.

When pharmacists without same-day naloxone were asked about alternative sources, approximately half provided no suggestion. Suggestions included directing callers to another pharmacy (45.84% [95% CI 37.77%-53.91%]), the public health department (3.87% [95% CI 0.48%-7.25%]), a syringe services program (0.06% [95% CI 0.00%-0.17%]), and other options (3.87% [95% CI 0.48%-7.25%]). Other suggestions included hospitals, libraries, public health boxes, Amazon, thrift stores, police stations, fire departments, web-based sites, and cannabis dispensaries.

### Cost of Naloxone

Information on cost was restricted to pharmacies offering same-day availability without a prescription. The mean (SE) cost of naloxone was $52.07 ($1.50) (95% CI, $49.12-$55.02).

### Type of Pharmacy Associated With Same-Day Naloxone Without a Prescription

Logistic regression analysis examined the association between pharmacy type and the availability of same-day naloxone without a prescription. Compared with corporate chains, food store and/or mass merchandisers (OR, 0.53 [95% CI, 0.35-0.82]), medical affiliates (OR, 0.23 [95% CI, 0.03-1.68]), and independent pharmacies (OR, 0.09 [95% CI, 0.05-0.16]) all had lower odds of same-day naloxone availability.

### Neighborhood Characteristics of Pharmacies

We determined that pharmacies located in areas with a greater percentage of White residents had greater odds (OR per 1–percentage point change, 1.13 [95% CI, 1.04-1.22]) of having naloxone available the same day without a prescription (Table). There were no statistically significant differences in the measures related to neighborhood disadvantage where pharmacies were located and same-day naloxone availability without a prescription.

**Table.** Association of Neighborhood Composition With Same-Day Naloxone Without a Prescription

Neighborhood composition, self-identified race and ethnicity^a^	Odds ratio (95% CI)	*P* value
Proportion identifying as American Indian or Alaska Native	0.25 (0.05-1.17)	.08
Proportion identifying as Black or African American	0.94 (0.85-1.05)	.27
Proportion identifying as Latino or Hispanic ethnicity of any race	0.24 (0.01-4.39)	.33
Proportion identifying as White race of any ethnicity	1.13 (1.04-1.22)	.003

^a^ Calculated as estimates per a change of 1 percentage point.

## Discussion

This cross-sectional study provides, to our knowledge, the first national assessment of naloxone availability, in-store placement, and pricing following its transition to OTC status. In this national sample, same-day naloxone without a prescription was available in more than 60% of the retail pharmacies, leaving nearly 40% of the locations without immediate access to naloxone. These findings highlight the progress toward accessibility to naloxone in retail pharmacies.

Despite the OTC designation, naloxone was most often kept behind the pharmacy counter, requiring customers to interact with pharmacy staff. This may limit visibility and create barriers for individuals who anticipate or have experienced stigma when seeking naloxone. A 2023 single-state study found that one of the most significant barriers to naloxone access was patient stigma-related factors.^23^ Implementing stocking and dispensing strategies for naloxone that allow customers to purchase the medication without interacting with a person at the pharmacy counter could potentially improve naloxone uptake.

Our findings indicate that contrary to initial concerns^24^ that naloxone cost would increase, the mean out-of-pocket cost for same-day access without a prescription was approximately $52.07. This aligns with recent data from North Carolina, where the mean cost decreased from $90.93 to $62.67 after the OTC transition.^14,18^ Another study conducted in the southern states of the US found that the median price of OTC naloxone was $50.00.^18^ These results suggest that the fears that the prices of naloxone would skyrocket or that there would be an anticipated surge in naloxone prices after OTC approval have not materialized. The cost of OTC naloxone still exceeds the retailer’s recommended price ($44.81).^19^ Therefore, policy efforts should focus on cost regulation, subsidies, and expanded insurance coverage. Pharmacies can improve affordability through price transparency and patient assistance programs.

Among pharmacies that did not have same-day availability of naloxone, callers asked where else they might obtain it. This moment offered an opportunity for pharmacists to serve as connectors to community-based resources. Prior research has shown that pharmacists are both trained and willing to engage in harm reduction within retail pharmacy settings.^25,26^ To do this effectively, pharmacists must be supported with clear, accessible information about local services. Our findings raise concern that syringe services programs, an evidence-based public health intervention, were the least frequently recommended option. Given that syringe services programs are not available in many communities,^27^ particularly in rural and southern areas, pharmacy referral tools should include a broad range of locally available resources rather than emphasizing any single referral option. Equipping pharmacists with updated referral tools could ensure they are empowered to guide individuals to resources when in-store access is unavailable.

This study highlights disparities in naloxone availability based on the type of pharmacy. Chain pharmacies are more likely to stock naloxone without a prescription compared with other pharmacy types, especially independent pharmacies. This discrepancy may be due to standardized policies that are common in chain pharmacies. Independent pharmacies, which often serve rural communities, may encounter challenges, including cost concerns and lower demand, resulting in less consistent stocking.^12,18^ Previous pharmacy studies have found that the ability to stock and dispense naloxone depended on pharmacy type.^16,28,29^ Targeted educational^12,18^ interventions and support for independent pharmacies could improve naloxone accessibility in underserved areas.

Our findings indicate that pharmacies located in census tracts with a higher percentage of White residents were more likely to offer same-day naloxone access. These findings suggest that inequities may exist not only in physical access to pharmacies but also in whether pharmacies are prepared to provide naloxone when individuals seek it. Addressing these disparities will require multifaceted strategies, including support for pharmacies serving racially and ethnically diverse communities, stronger referral networks, pharmacist training, and policy initiatives that incentivize consistent stocking and dispensing of OTC naloxone in underserved areas. These findings should be interpreted as reflecting naloxone availability among retail pharmacies rather than access across all neighborhood contexts. This distinction is important because equitable OTC naloxone access depends not only on whether pharmacies stock naloxone, but on whether pharmacies are present, accessible, and prepared to serve communities with the greatest need.

### Limitations

This study has several limitations. Secret shopper methods capture a single point in time and may not reflect all customer experiences or variability in staff responses. Callers spoke with 1 pharmacy staff member per pharmacy, and availability was assessed by telephone rather than verified in person; therefore, findings may reflect staff-reported availability rather than actual on-site product availability. Neighborhood sociodemographic measures at the census block group and tract levels may not capture individual-level disparities. Because naloxone access continues to evolve, findings should be interpreted as a snapshot of pharmacy access during the data collection period from January to April 2024. The study was not designed to estimate state-level variation. Future research should examine state-level differences, longitudinal trends, and qualitative factors influencing OTC naloxone implementation in retail pharmacies and to examine how pharmacist-identified barriers and facilitators to OTC naloxone implementation can be translated into a pharmacy-adapted overdose prevention intervention that improves equitable naloxone access across pharmacy settings.

## Conclusions

In this national cross-sectional study, same-day naloxone availability and price following OTC approval remained uneven across pharmacy types and community demographics. Although costs had not increased dramatically, prices still exceeded the retailer’s recommended level, potentially limiting affordability for individuals facing financial constraints. Expanding pharmacist training, targeted pharmacy support, price transparency, insurance coverage, and subsidy programs may strengthen naloxone affordability and access.

## Notes

Supplement 1. **eAppendix.** Disposal Mystery Caller Data Collection

Supplement 2.
Data Sharing Statement
