# Supplementary material for: Availability of Naloxone in Retail Pharmacies Following Introduction of Over-the-Counter Status
Source: JAMA Netw Open. 2026 Jul 17;9(7):e2623617. doi: 10.1001/jamanetworkopen.2026.23617 (PMC13379737; doi:10.1001/jamanetworkopen.2026.23617)
Supplement: Supplement 1. — eAppendix. Disposal Mystery Caller Data Collection [file jamanetwopen-e2623617-s001.pdf]

Supplementary Online Content

Eldridge LA, Kline D, Fields K, et al. Availability of naloxone in retail pharmacies following introduction of over-the-counter status. *JAMA Netw Open.* 2026;9(7):e2623617. doi:10.1001/jamanetworkopen.2026.23617

eAppendix. Disposal Mystery Caller Data Collection

This supplementary material has been provided by the authors to give readers additional information about their work

© 2026 Eldridge LA et al. *JAMA Network Open*.

eAppendix. Disposal Mystery Caller Data Collection

Record ID

____

INSTRUCTIONS:

Before you make the call -

1) Add the pharmacy ID number from the Excel Spreadsheet.
2) Add the phone number associated with the pharmacy ID from the Excel Spreadsheet.
3) Add your mystery caller ID.

Pharmacy ID

____

Phone Number

____

Mystery Caller ID

____

INSTRUCTIONS:

Now, you are ready to make the call. A few notes before you dial -

*Wait at least 1 minute for someone to answer your call. If no one answers after 1 minute, end the attempt.
*If they put you on hold, end the attempt after 4 minutes of waiting.
*If they offer to call you back, politely decline.

Make sure to record details at about your attempted call towards the end of this data collection form. We will make up to three attempts for each pharmacy on different days.

About your role: You are someone with a few pills remaining after surgery for a broken arm.

"Hi, I have some Vicodin that I need to get rid of. I heard some pharmacies will take back medications or give you something to dispose of them at your home. Does your pharmacy do this?"

- ☐ No
- ☐ Yes, but no additional information was provided
- ☐ Yes, give it directly to the pharmacist
- ☐ Yes, disposal box
- ☐ Yes, other option

If they provided a response that was not an option for you, summarize the response here.

____

"Where is the box located in the store?"

- ◯ Near the pharmacy counter
- ◯ Near the front checkout
- ◯ Other

Where is the box located?

____

© 2026 Eldridge LA et al. *JAMA Network Open*.

If they say no to having disposal options at the pharmacy:<br><br>"Ok. How would you recommend that I get rid of my Vicodin?"	☐ No other option provided<br>☐ A different pharmacy<br>☐ Law enforcement agency<br>☐ Hold on to them for a take-back event<br>☐ Mix them with an undesirable substance (they may state one) and put it in the trash<br>☐ Flush them down the toilet or sink<br>☐ Use a deactivation product<br>☐ Use a mail back envelope<br>☐ Other
Specify other ways to dispose of medication	__________________________________
Please include any other noteworthy questions or statements made by the pharmacist/pharmacy staff.	__________________________________
Did the pharmacy staff member reveal their current position?	◯ Yes<br>◯ No
What was their role?	◯ Pharmacist<br>◯ Pharmacy assistant<br>◯ Pharmacy technician<br>◯ Other

INSTRUCTIONS:

The remaining section is to record information about your call attempt(s).

Date of Call (1)	____________________________<br>(MM/DD/YYYY)
Time of Call (1)	____________________________<br>(HH:MM (am/pm))
Were you able to speak with a pharmacy staff member about disposal options? (1)	◯ No<br>◯ Yes<br>(no--> end call)
Date of Call (2)	____________________________<br>(MM/DD/YYYY)
Time of Call (2)	____________________________<br>(HH:MM (am/pm))
Were you able to speak with a pharmacy staff member about disposal options? (2)	◯ No<br>◯ Yes<br>(no--> end call)
Date of Call (3)	____________________________<br>(MM/DD/YYYY)

Time of Call (3)	______________________<br>(HH:MM (am/pm))
Were you able to speak with a pharmacy staff member about disposal options? (3)	◯ No<br>◯ Yes<br>(no--> end call)
Was this pharmacy still in business?	◯ Yes<br>◯ No
Does this pharmacy serve community members (assume yes unless they tell you otherwise)?	◯ Yes<br>◯ No
