# Supplementary material for: Availability of Naloxone in Retail Pharmacies Following Introduction of Over-the-Counter Status
Source: JAMA Netw Open. 2026 Jul 17;9(7):e2623617. doi: 10.1001/jamanetworkopen.2026.23617 (PMC13379737; doi:10.1001/jamanetworkopen.2026.23617)
Supplement: Supplement 2. — Data Sharing Statement [file jamanetwopen-e2623617-s002.pdf]

Data Sharing Statement

Eldridge. Availability of Naloxone in Retail Pharmacies Following Introduction of Over-the-Counter Status. *JAMA Netw Open*. Published July 17, 2026.
doi:10.1001/jamanetworkopen.2026.23617

Data
Data available: Yes
Data types: Deidentified participant data, Data dictionary
How to access data: Data will be available upon formal request to the lead author.
When available: With publication

Supporting Documents
Document types: Statistical/analytic code
How to access documents: Information will be available upon formal request to the lead author.
When available: With publication

Additional Information
Who can access the data: NA
Types of analyses: Information will be made available when a specified purpose is provided to the lead author.
Mechanisms of data availability: Data will be shared after a signed data access agreement has been received.
Any additional restrictions: NA
